# Online Delivery of Interprofessional Adverse Childhood Experiences Training to Rural Providers: Usability Study

**DOI:** 10.2196/56722

**Published:** 2024-08-07

**Authors:** Julie M Kapp, Rachel Dicke, Kathleen Quinn

**Affiliations:** 1 Department of Public Health, College of Health Sciences, University of Missouri, 806 Lewis Hall, Columbia, MO, 65201, United States, 1 5738843684; 2 Institute of Public Policy, Truman School of Government and Public Affairs, University of Missouri, Columbia, MO, United States; 3 School of Medicine, University of Missouri, Columbia, MO, United States

**Keywords:** adverse childhood experiences, ACE, training, trauma-informed care, provider, rural, adverse, trauma, traumatic, provider, providers, teaching, curriculum, curricula, education, educational, social work, social worker, social workers, psychologist, psychologists, counselor, counselors, interprofessional, pediatric, pediatrics, paediatric, paediatrics, child, children, experience, experiences, continuing education

## Abstract

**Background:**

The population health burden of adverse childhood experiences (ACEs) reflects a critical need for evidence-based provider training. Rural children are also more likely than urban children to have any ACEs. A large proportion of providers are unaware of the detrimental effects of ACEs. There is a significant documented need for training providers about ACEs and trauma-informed care, in addition to a demand for that training.

**Objective:**

The objective was to develop, implement, and evaluate an online ACEs training curriculum tailored to Missouri providers, particularly those in rural areas given the higher prevalence of ACEs.

**Methods:**

From July 2021 to June 2022, we conducted literature reviews and environmental scans of training videos, partner organizations, clinical practice guidelines, and community-based resources to curate appropriate and tailored content for the course. We developed the ACEs training course in the Canvas learning platform (Instructure) with the assistance of an instructional designer and media designer. The course was certified for continuing medical education, as well as continuing education for licensed professional counselors, psychologists, and social workers. Recruitment occurred via key stakeholder email invitations and snowball recruitment.

**Results:**

Overall, 135 providers across Missouri requested enrollment, with 72.6% (n=98) enrolling and accessing the training. Of the latter, 49% (n=48) completed course requirements, with 100% of respondents agreeing that the content was relevant to their work, life, or practice; they intend to apply the content to their work, life, or practice; they feel confident to do so; and they would recommend the course to others. Qualitative responses supported active intent to translate knowledge into practice.

**Conclusions:**

This study demonstrated the feasibility, acceptability, and effectiveness of interprofessional workforce ACEs training. Robust interest statewide reflects recognition of the topic’s importance and intention to translate knowledge into practice.

## Introduction

A large proportion of providers are unaware of the detrimental effects of adverse childhood experiences (ACEs) [1]. ACEs are commonly defined as personal abuse that is psychological, physical, or sexual; personal neglect that is emotional or physical; or household challenges that include violence against the mother, divorce, or separation; or living with household members who were substance abusers, mentally ill or suicidal, or had ever been imprisoned [2,3]. Other studies consider additional adversities such as the death of a parent or sibling, poor housing, discrimination, bullying, and poverty, among others [4,5].

If we can reduce intergenerational transmission of ACEs, we have the potential to mitigate ACEs and theoretically make profound improvements in population health outcomes. Overall, 60.9% of US adults experience 1 or more than 1 type of ACE and 15.6% experience 4 or more than 4 types [6]. ACEs increase the risk of adult diseases including heart disease, cancer, chronic lung disease, skeletal fractures, and liver disease via chronic stress and coping behaviors [2,6]. Cumulative exposures to multiple ACEs are associated with an increased risk of substance misuse [2,7,8], including opioids [9-11] and alcohol [12], and an increased risk of suicide [2]. Suicide and other “deaths of despair,” ie, deaths from suicide and drug poisoning (including opioids and alcohol) contribute significantly to rising US mortality rates [13]. A 10% reduction in the prevalence of ACEs equates to an annual savings of 3 million disability-adjusted life years or $105 billion US [14]. Illicit drug use had the highest population-attributable fractions associated with ACEs, and population-attributable fractions of causes of ill health were highest for mental illness outcomes: ACEs were attributed to about 30% of cases of anxiety and 40% of cases of depression in North America [14]. The intersection of ACEs, suicide, and overdose is a priority area for the United States Department of Agriculture [15] and the Centers for Disease Control and Prevention (CDC) [16]. Finally, an objective of the US Department of Health and Human Services’ Healthy People 2030 is to reduce the number of young adults (aged 18 to 25 years) who report 3 or more than 3 ACEs; this is described as a high-priority public health issue with evidence-based interventions to address it [17].

In addition to the rising US mortality rates mentioned above, there is a place-based mortality penalty, especially for high-poverty rural areas [18]. Rural children are statistically more likely than urban children to experience economic hardship, household substance misuse, household mental illness, household incarceration, and parental separation or divorce, as well as witness neighborhood violence and household violence [19]. Rural children are also more likely than urban children to have any ACEs [19].

There is a significant documented need for training providers about ACEs and trauma-informed care, in addition to a demand for that training [1]. Practical considerations exist with the implementation of training, including time and institutional support, interactive components, practical examples, and not retraumatizing providers who have themselves experienced ACEs [1]. A commentary in 2020 by Campbell [20], suggested that there could be harm to patients from ACEs screening, despite acknowledging there is an evidence base for related clinical preventive services, such as screening for alcohol and drug use disorder and interpersonal violence. A response to this caution coauthored by the investigator of the original ACEs study Felitti stated that “ACEs screening was used as part of a comprehensive health assessment at Kaiser in more than 440,000 adult patients without ever evoking a complaint” [21] and recommended to implement ACEs screening in practice with enthusiasm. Still, the identification of ACEs is only 1 component of translating the science into practice. Providers need to be ready with a planned intervention after ACEs are identified, depending upon a primary, secondary, or tertiary prevention response. Finally, providers need to be aware of positive childhood experiences so they can recognize and encourage these behaviors in parents.

Evaluating the uptake, satisfaction, and effectiveness of ACEs trainings on provider practice is key [1]. The purpose of this project was to develop, implement, and evaluate an online workforce training curriculum tailored to Missouri providers (particularly rural) to improve their capacity to provide evidence-based care to high-needs patient populations in resource-limited settings.

## Methods

### Setting

Missouri ranks 34th nationwide on ACEs, with 17% of children aged 0‐17 years having experienced 2 or more ACEs [22]. In Missouri, 99 out of the 115 (86.1%) counties (114 counties plus the city of St. Louis) are rural, and 33.2% of Missouri’s population lives in rural counties [23].

### Participants

This study is part of a larger project with the overall goal of training physicians who work in rural communities and providing them with the highest level of up-to-date information. Our training was tailored toward interdisciplinary Missouri providers, with the goal of reaching a significant proportion of rural providers. All provider types (including physicians, clinicians, professionals, paraprofessionals, and ancillary service staff) were eligible to enroll so the course would reflect a cross-sector approach and participants could learn from each other in the discussions. We were particularly interested in enrolling rural clinicians. A separate paper details the evaluation of the discussion posts [24].

### Course Development

From July 2021 to June 2022, we conducted literature reviews and environmental scans of training videos, partner organizations, clinical practice guidelines, and community-based resources to curate appropriate and tailored content for the course. The content of the course included but was not limited to select published works from Felitti, ACEs videos developed by the CDC [25,26], a TED talk by Harris [27] on how childhood trauma affects health across the lifespan, US Preventive Services Task Force recommendations for screening, and other literature. Resources included but were not limited to Findhelp, Missouri Department of Health and Senior Services’ crime victim services, home visiting programs, related partner organizations, screening, and measurement tools. We developed the ACEs training course in the Canvas learning platform with the assistance of an instructional designer and media designer. The designers played a key role in style, usability, design, and best practices for online learning. The course was designed to be asynchronous with interactive elements, such as videos, discussions, and quizzes. Given providers may themselves have experienced childhood adversity, a trigger warning was included on the home page and a mindfulness breathing exercise was included in the instructions.

We pilot-tested the course with health provider colleagues for content, gaps, length, satisfaction, and usability prior to implementation. The final course outline is described in Textbox 1.

Textbox 1. **Adverse childhood experiences course outline.**

**Module 0: Getting started**

- This module includes a welcome, introduction of the instructor, demographic survey for participants to complete, an overview of the course and expectations, and a discussion.

**Module 1: What are adverse childhood experiences?**

- This module includes an overview of the module and explanation of the original adverse childhood experiences (ACEs), expanded ACEs, a discussion, and a quiz.

**Module 2: How do adverse childhood experiences impact health?**

- This module includes an overview of the module and explanation of ACEs’ impact on the developing brain, adult health, depression, substance use disorders, other impacts, increased risk of disease, and population-attributable risk. The module includes a discussion and quiz.

**Module 3: How common are adverse childhood experiences?**

- This module includes an overview of the module and explanation of how common ACEs are in the United States, Missouri, rural areas, and the status of Missourians’ health and wellness outcomes. The module includes a discussion and quiz.

**Module 4: How did the COVID-19 pandemic affect adverse childhood experiences?**

- This module includes an overview of the module and explanation of the psychological impact of the COVID-19 pandemic, as well as how the COVID-19 pandemic affected ACEs. The module includes a discussion and quiz.

**Module 5: What can we do to address adverse childhood experiences?**

- This module includes an overview of the module and explanation of the risk factors for ACEs, what can be done at the provider level, what the evidence says about screening, positive childhood experiences and protective factors, what can be done at the organizational, community, and policy levels. The module includes a discussion and quiz.

**Module: Wrap up**

- This module includes links to resources, explanation of how to enroll in the ACEs listserv, and instructions for completing the course and course evaluation.

### Certification

As an incentive to enroll and complete the training, we offered continuing education (CE) credits free of charge to participants. The office of Continuing Education for Health Professions (CEHP) is housed on the University of Missouri campus. CEHP’s mission is to provide evidence-based, relevant, and responsive learning activities designed to narrow professional practice gaps with respect to the knowledge, competence, and performance of the health care team.

CEHP reviewed the course content for continuing medical education credits, awarding the course 3 American Medical Association Physician's Recognition Award Category 1 Credits. Upon request from enrollees, the office also reviewed the course for CE for licensed professional counselors, psychologists, and social workers with Missouri licenses. CEHP attested that this course contains 3 clock hours of instructional time. Licensed professionals measuring CE credit based on a 50-minute hour may claim up to 3.6 contact hours for full attendance at this program. Certificates of completion were also available.

To qualify for CE credit or a certificate of completion, trainees were required to complete (1) a demographic survey required by the funder and available in the introductory course pages; (2) six discussion assignments; (3) five quizzes, receiving a grade of at least 80% on each; and (4) the end-of-course evaluation. The course evaluation is linked to a separate page in Qualtrics (Qualtrics, LLC). We chose to make the course evaluation anonymous to protect the respondents’ identity and to improve the integrity of the responses while recognizing we would not be able to individually validate trainees’ completion of this course requirement. Some of the demographic survey questions (eg, do you consider yourself disadvantaged or the level of rurality of their hometown setting) are reflective of self-identity and self-report rather than objective indicators.

### Recruitment, Enrollment, and Tracking

The course was launched via key stakeholder email invitations in late November 2022 with snowball recruitment thereafter. Email invitations included a statewide listserv focused on responding to the opioid crisis, as well as to a network of rural providers. The recruitment email included that the course is an asynchronous online course available through June 30, 2023, free of charge to participants, offers free CE credit, and a basic course outline. Interested individuals sent an email with a request to enroll in the training. The individual was then sent instructions for creating a Canvas account, with technical support available for troubleshooting challenges. Once the Canvas account was created, the individual was subsequently invited into the ACEs training. Course requests, enrollment, and completion were tracked in Microsoft Excel. Periodically throughout the course implementation period, study personnel sent email reminders to those enrolled regarding the end date for the training.

### Data Analyses

The data used for analysis in this study included the course enrollment tracking data, the demographic survey data, and the course evaluation survey data. Frequencies and percentages were calculated for quantitative data; missing responses were not included in the calculations. Age was approximated by subtracting birth year collected in the sponsor demographic survey from 2023. Short-answer open-ended questions were qualitatively coded into major themes independently by 2 authors and then reviewed for divergence. Reported quotes were lightly copyedited.

### Ethical Considerations

The University of Missouri Institutional Review Board reviewed and approved this project (#2096686).

## Results

### Enrollment and Completion

Overall, 135 individuals requested enrollment into the ACEs training. Of those, 98 (72.6%) completed the steps for enrollment and accessed the course. Among the individuals who enrolled and accessed the course, 70 (71.4%) completed the demographic survey, and 48 (49%) completed all course requirements.

### Participant Characteristics

The setting in which participants worked varied (Table 1). The majority of participants worked in “other” settings (n=28, 40%), followed by clinical sites (n=12, 17.1%); academic medical centers (n=8, 11.4%); federally qualified health centers or Look-Alikes (n=6, 8.6%; Look-Alikes are community-based health center programs that meet program requirements but do not receive health center program funding); government (n=6, 8.6%); private practice or industry (n=4, 5.7%); and public community, rural, or migrant health centers (n=4, 5.7%). Responses for the employment setting location included 27.1% (n=19) rural areas, 25.7% (n=18) health professional shortage areas, 18.6% (n=13) medically underserved communities, and 12.9% (n=9) primary care settings. Most (n=30, 42.9%) participants reported their hometown setting as rural, with 34.3% (n=24) suburban and 18.6% (n=13) urban.

Regarding participant characteristics, 84.3% (n=59) reported their gender as female, with a balanced distribution across age groups 20 to 59 years. The majority of participants reported White race (n=62, 88.6%), and a minority (n=11, 15.7%) considered themselves disadvantaged (defined as growing up in an area that was medically underserved or had insufficient access to social, economic, and educational opportunities). Respondents’ professional disciplines consisted of 50% (n=35) behavioral health, 18.6% (n=13) nursing, 14.3% (n=10) other, 10% (n=7) public health, 4.3% (n=3) student, and 2.9% (n=2) medicine. Enrollee fields and degrees included bachelors (BS, BA, and BSPH), nursing (RN, FNP, BSN, DNP, and CPNP), social work (MSW, LMSW, and LCSW), licensed professional counselors (MA, MAC, MS, MEd, and PLPC), others with master’s degrees (MPH, MS, and MPA), doctorates (PhD), and physicians (MD).

**Table 1.** Characteristics of adverse childhood experiences course participants who completed the demographic survey (n=70).

Variable			Values, n (%)
**Personal attributes**			
	**Gender**		
		Female	59 (84.3)
		Male	8 (11.4)
		Nonbinary	1 (1.4)
	**Age groups (years)**		
		20‐29	16 (22.9)
		30‐39	18 (25.7)
		40‐49	14 (20.0)
		50‐59	17 (24.3)
		60‐69	3 (4.3)
	**Hispanic, Latino/a/x, or Spanish origin**		
		Yes	0 (0)
		No	68 (97.1)
	**Race**		
		American Indian or Alaskan Native	0 (0)
		Asian	2 (2.9)
		Black or African American	3 (4.3)
		White	62 (88.6)
	**Hometown setting**		
		Rural	30 (42.9)
		Suburban	24 (34.3)
		Urban	13 (18.6)
	**Do you consider yourself disadvantaged?** ^**a**^		
		Yes	11 (15.7)
		No	56 (80.0)
	**Professional discipline**		
		Behavioral health	35 (50.0)
		Medicine	2 (2.9)
		Nursing	13 (18.6)
		Public health	7 (10.0)
		Student	3 (4.3)
		Other	10 (14.3)
**Employment attributes**			
	**Employment setting (check all that apply)**		
		Academic medical center	8 (11.4)
		Area health education center	2 (2.9)
		Federally qualified health center or Look-Alike	6 (8.6)
		Government	6 (8.6)
		Nonacademic medical center	1 (1.4)
		Other clinical site	12 (17.1)
		Other health center	2 (2.9)
		Private practice or industry	4 (5.7)
		Public setting—community, rural, or migrant health center	4 (5.7)
		Public setting—health department	2 (2.9)
		Rural health clinic	1 (1.4)
		Other	28 (40.0)
	**Employer is located in (check all that apply)**		
		Health professional shortage area	18 (25.7)
		Medically underserved community	13 (18.6)
		Primary care setting	9 (12.9)
		Rural area	19 (27.1)

a You might consider yourself disadvantaged if you grew up in an area that was medically underserved or had insufficient access to social, economic, and educational opportunities.

### Course Evaluation

Of those who completed the training, 42 (87.5%) completed the course evaluation. Quantitative responses are summarized in Table 2. Almost three-fourths of respondents live, work, or serve in rural areas of Missouri.

All respondents (100%) agreed or strongly agreed that the content is relevant to their work, life, or practice; the learning objectives are clear; the resources are helpful; they intend to apply the course content to their work, life, or practice; they feel confident implementing the knowledge gained; and they would recommend the course to others. Most respondents agreed or strongly agreed the length is appropriate (n=41, 97.6%), the videos are informative (n=41, 97.6%), the quizzes provide a helpful self-assessment (n=40, 95.2%), and the discussions are valuable (n=33, 78.6%). Most respondents rated the course overall as excellent or very good (n=39, 92.9%).

A total of 37 respondents answered the question “Please describe the strengths of the course, ie, elements you liked, things that worked well, ‘a-ha’ moments, helpful course content or features.” Responses included a breadth of course components—videos, rural or urban comparisons, statistics, discussion, quizzes, charts or graphs, resources, and online format. Eleven respondents provided suggestions to the question “Please describe opportunities to improve the course, ie, challenges with navigating the course, missing content, barriers to completing the course, etc.” Three respondents did not care for the discussion boards, 2 had technology problems, 1 did not care for the quizzes, and 1 felt the online format was limiting the interaction. The remaining suggestions included incorporating brainstorming technology such as “Jam Boards,” more videos on how to implement the research in the workplace, more emphasis on the role of social workers, and case studies asking people how they would approach a scenario.

**Table 2.** Results of adverse childhood experiences course evaluation quantitative questions (n=42).

Question name and response option		Values, n (%)
**The content is relevant to my work, life, or practice**		
	Strongly agree or agree	42 (100.0)
	Neutral or not sure	0 (0.0)
	Strongly disagree or disagree	0 (0.0)
**The learning objectives are clear**		
	Strongly agree or agree	42 (100.0)
	Neutral or not sure	0 (0.0)
	Strongly disagree or disagree	0 (0.0)
**The length is appropriate**		
	Strongly agree or agree	41 (97.6)
	Neutral or not sure	1 (2.4)
	Strongly disagree or disagree	0 (0.0)
**The videos are informative**		
	Strongly agree or agree	41 (97.6)
	Neutral or not sure	1 (2.4)
	Strongly disagree or disagree	0 (0.0)
**The quizzes provided a helpful self-assessment**		
	Strongly agree or agree	40 (95.2)
	Neutral or not sure	1 (2.4)
	Strongly disagree or disagree	1 (2.4)
**The discussions are valuable**		
	Strongly agree or agree	33 (78.6)
	Neutral or not sure	8 (19.0)
	Strongly disagree or disagree	1 (2.4)
**The resources are helpful**		
	Strongly agree or agree	42 (100.0)
	Neutral or not sure	0 (0.0)
	Strongly disagree or disagree	0 (0.0)
**I intend to apply the course content in my work, life, or practice**		
	Strongly agree or agree	42 (100.0)
	Neutral or not sure	0 (0.0)
	Strongly disagree or disagree	0 (0.0)
**I feel confident that I can implement the knowledge I gained**		
	Strongly agree or agree	42 (100.0)
	Neutral or not sure	0 (0.0)
	Strongly disagree or disagree	0 (0.0)
**Please rate the course overall**		
	Excellent or very good	39 (92.9)
	Good	3 (7.1)
	Fair or poor	0 (0.0)
**I would recommend this course to others**		
	Strongly agree or agree	42 (100.0)
	Neutral or not sure	0 (0.0)
	Strongly disagree or disagree	0 (0.0)
**Do you live, work in, or serve individuals in rural areas of Missouri?**		
	Yes	31 (73.8)
	No	11 (26.2)

### Translation to Practice

A total of 33 respondents answered the question “If you intend to apply the course content to your work, life, or practice, please describe how,” with 26 (78.8%) respondents reporting some level of active intent and 14 (42.4%) respondents suggested using the course content for active organizational planning or changing their practice toward a more advanced trauma-informed care work environment.

*Apply to current and future practice in the health care field with children and adults*.

[Example response 1]

*I want to keep in mind the mother’s ACE score when working with them in parenting classes. Seeing how their own ACE scores affect them and how they can affect their children*.

[Example response 2]

*Moving our organization past trauma awareness and into more trauma-informed work.*

[Example response 3]

*I will look to implement ACEs screening in our organization and increase trauma informed care.*

[Example response 4]

*I work with youth in a school setting. This could be helpful to implement trauma informed care in the school setting, while also being aware of ACEs with clients.*

[Example response 5]

*We are currently working on ways to prevent overdose events among youth and have been considering how we can take ACEs into account in preventing overdose and long-term negative health effects related to addiction.*

[Example response 6]

*This information is very important to my work with people as well in life. Also, to my organization as we continue to grow towards a trauma-informed organization.*

[Example response 7]

*Information will help inform TIC team in evaluating policies.*

[Example response 8]

A total of 8 (24.2%) respondents suggested the training will help them actively educate others.

*Spreading the word on the existence of ACEs and how to use them for positive growth in children.*

[Example response 1]

*Psychoeducation in therapy.*

[Example response 2]

*Educating my organization and other community partners.*

[Example response 3]

*Educating youth and families.*

[Example response 4]

Also, 4 (12.1%) respondents reported an intent to screen for ACEs.

*Have begun utilizing ACEs scoring with incarcerated population.*

[Example response 1]

*I work...with clients in the mental health field, so completing the ACEs when they enroll will provide us with more history of the clients and their experiences.*

[Example response 2]

*I can use this as I get to know my students. I plan to start using the ACE test as I assess how best I can help my students.*

[Example response 3]

Finally, 7 (21.2%) alluded to awareness, which was coded as a passive reaction to applying the course content. Example quotes included:

*I find it important to be aware of ACEs as a counselor in learning to understand why and how people present as they do in a college setting.*

[Example response 1]

*I am aware of the need for more primary care providers to have training in this area.*

[Example response 2]

*I work with foster/adoptive families and ACEs are big part of their lives.*

[Example response 3]

*I have a new list of resources to utilize, and a better knowledge of what ACEs are and what they might look like in a school setting.*

[Example response 4]

*My knowledge of...COVID-19’s effects on ACEs, and [the heightened level of ACEs in rural areas] keeps me more aware of key things to look for.*

[Example response 5]

## Discussion

### Principal Findings

A limited body of literature exists related to educating providers on ACEs and trauma-informed care, as well as evaluating their knowledge, perceived importance, and attitudes toward ACEs in practice [1]. The objective of our study was to develop, deliver, and evaluate an interprofessional, online, asynchronous ACEs training program for interdisciplinary providers across Missouri, especially in rural areas. With a large proportion of providers unaware of the detrimental effects of ACEs, significant need for training providers about ACEs and demand for that training [1], our findings add critical insight to the uptake, satisfaction, and effectiveness of ACEs provider training. Our study additionally addresses a critical gap with its focus on rural providers. Increasing the number of ACEs and trauma-aware providers is a step toward increasing trauma-informed care. The Substance Abuse and Mental Health Services Administration [28] considers being trauma-informed as having a basic realization of trauma and how it impacts families and individuals, recognizing the signs of trauma, responding by applying the principles of a trauma-informed approach, and resisting retraumatizing individuals. These four assumptions work in conjunction with their six key principles and they are (1) safety; (2) trustworthiness and transparency; (3) peer support; (4) collaboration and mutuality; (5) empowerment, voice, and choice; and (6) cultural, historical, and gender issues. The Substance Abuse and Mental Health Services Administration further includes cross-sector collaboration among its implementation practices; an additional strength of our training includes its interprofessional scope of providers.

The overall response to recruitment efforts was stronger than anticipated given we leveraged only a few key communication channels, with 135 individuals statewide requesting enrollment. Additionally, our recruitment and enrollment effort successfully reached the target demographic, with 73.8% (n=31) of participants living, working, or serving in rural areas, despite the broadband challenges. While 90% of rural Missouri counties have broadband internet, Missouri ranks 43rd among states in internet coverage speed and availability [23]. In Missouri, 41 rural counties do not have a hospital [23], making ACEs awareness efforts at the prevention level even more crucial. The course was designed as an asynchronous, online course and technology can sometimes be a barrier, particularly for age cohorts educated prior to the popularity of online courses. However, while the age group distribution for those who completed enrollment was nicely balanced across 20 to 59 years of age, it is possible a selection bias occurred with the 27.4% of individuals who requested enrollment but did not complete enrollment being less comfortable with technology, despite the available assistance. Finally, enrollment by medical providers was modest, despite the asynchronous format to accommodate busy schedules and available continuing medical education.

Among participants completing the course, the course evaluation indicated strong levels of satisfaction with the structure and content. This is particularly encouraging given the interdisciplinary nature of the content, as it was not tailored to a specific discipline by design, yet 100% of respondents agreed the content was relevant to their work, life, or practice; that they intend to apply the content to their work, life, or practice; they feel confident to do so; and would recommend the course to others. Regarding what could be improved, respondents scored the value of the discussions lowest compared to other content questions (78.6% agreed or strongly agreed the discussions are valuable). We attribute this score to the discussion requiring the most active engagement on the part of participants. Active learning is a best practice in online learning, as well as a requirement for these CE credits.

Among the respondents who reported intent to apply the course content in practice, 78.8% suggested active planning, training, or screening, naming specific examples. This is an encouraging indication of effective knowledge transfer and declaration of intention.

### Conclusions

This study developed, implemented, and evaluated an online workforce training curriculum tailored to Missouri providers (particularly rural) to improve their capacity to provide evidence-based care to high-needs patient populations in resource-limited settings. This study demonstrated the feasibility, acceptability, and effectiveness of interprofessional online workforce training of ACEs, while also reaching resource-limited rural counties. Robust interest statewide reflects recognition of the topic’s importance and the potential for this training to effect change. Future research is needed to follow up with participants to inquire about knowledge retention and actual implementation in practice, as well as ultimately any benefit to the patient.

## Abbreviations

ACE: adverse childhood experience
CDC: Centers for Disease Control and Prevention
CE: continuing education
CEHP: Continuing Education for Health Professions
